# Multidisciplinary Management of Pituitary Neuroendocrine Tumors During Pregnancy: Institutional Report

**DOI:** 10.1155/ije/1854208

**Published:** 2025-05-20

**Authors:** Zhiyuan Xiao, Xiaopeng Guo, Yong Yao, Jifang Liu, Wei Lian, Kan Deng, Yu Zhang, Xia Zhang, Yuanli Zhao, Yi Zhang, Bing Xing, Huijuan Zhu

**Affiliations:** ^1^Department of Neurosurgery, Peking Union Medical College Hospital, Chinese Academy of Medical Sciences and Peking Union Medical College, Beijing, China; ^2^Department of Neurosurgery, Key Laboratory of Endocrinology of Ministry of Health, China Pituitary Adenoma Specialist Council, Peking Union Medical College Hospital, Chinese Academy of Medical Sciences and Peking Union Medical College Hospital, Beijing 100730, China; ^3^Department of Neurosurgery, Medical Research Center, State Key Laboratory of Complex Severe and Rare Diseases, Peking Union Medical College Hospital, Chinese Academy of Medical Sciences and Peking Union Medical College Hospital, Beijing 100730, China; ^4^PUMCH Pituitary Health Innovation Center, Peking Union Medical College Hospital, Chinese Academy of Medical Sciences and Peking Union Medical College Hospital, Beijing 100730, China; ^5^Department of Anesthesiology, Peking Union Medical College Hospital, Chinese Academy of Medical Sciences and Peking Union Medical College Hospital, Beijing 100730, China; ^6^Department of Ophthalmology, Peking Union Medical College Hospital, Chinese Academy of Medical Sciences and Peking Union Medical College Hospital, Beijing 100730, China; ^7^Department of Endocrinology, Peking Union Medical College Hospital, Chinese Academy of Medical Sciences and Peking Union Medical College Hospital, Beijing 100730, China

**Keywords:** MDT, multidisciplinary team, pituitary neuroendocrine tumors, pregnancy, transsphenoidal surgery

## Abstract

**Background:** Management of pituitary neuroendocrine tumors (pitNETs) during pregnancy is challenging. Involvement of the multidisciplinary team (MDT) may benefit the collaborative decision-making. However, this aspect has not been well documented. We provided cases with pitNETs during pregnancy and summarized our experience on the MDT-guided management.

**Methods:** We performed a retrospective study enrolling all pregnant patients with pitNETs treated at our institute between March 1995 and July 2024.

**Results:** During the indexed period, 121 patients with pitNETs consulted our institute during pregnancy, with 111 of them being treated conservatively and 10 undergoing surgery due to progressive visual defect and other symptoms. The age of the included surgical cases was 33 years, and the gestational session at surgery ranged from 13 to 36 weeks (1 in the first trimester, 4 in the second, and 5 in the third). Of the resected tumors, six were nonfunctioning and the other four were functioning (1 lactotroph, 1 somatotroph, and 1 thyrotroph). All surgical cases received MDT-guided management by physicians from neurosurgery, endocrinology, ophthalmology, obstetrics, pediatrics, and anesthesiology, leading to gross total tumor resection, improved visual acuity, and successful delivery in all patients.

**Conclusion:** MDT guided management is essential for pitNETs during pregnancy. Surgical tumor resection is necessary for patients whose symptoms deteriorate rapidly. Transsphenoidal operation under general anesthesia is safe for pregnant patients with pitNETs.

## 1. Introduction

Pituitary neuroendocrine tumors (pitNETs) is defined as a clonal neoplastic proliferation of the anterior pituitary hormone-producing cells [1]. They occur with a mean incidence of approximately 5.1 cases per 100,000 annually and accounts for 23.42% of all primary brain tumors, making it the second most common tumor after meningiomas [2, 3]. The frequency of types is as follows: nonfunctional is at 57% and functional is at 43% (including: growth hormone [GH]-producing; prolactin [PRL]-producing, adrenocorticotrophic hormone [ACTH]-producing, gonadotropin-producing, GH-PRL-producing, and thyroid-stimulating hormone [TSH]-producing) [4]. Symptoms of pitNETs may be due to the hormones produced by the tumor or to pressure drainage to nearby structures. Tumor compression on the optic nerve causes visual acuity deterioration and visual field deficits. Compression of the normal pituitary gland can cause hypopituitarism. Hyperprolactinemia could also be caused by the compression of the pituitary stalk [5].

However, young women may find themselves in one of two situations prior to becoming pregnant. The first is awareness of a pitNETs, while the second is lack of awareness of the same condition. As the tumor grows, pregnancy can cause enlargement of pre-existing pitNET, which can compress the meninges and contribute to acute symptoms such as severe headache and visual disturbances. In some cases, combined with stroke or ischemic changes, this can lead to decreased vision and visual field loss. In severe cases, this can lead to complete blindness in both eyes [6]. In these cases, the choice between surgery or conservative treatment is challenging. This is an important clinical issue, particularly challenging for the neurosurgeon in terms of timing of surgery, indications, anesthetic risks, and maternal and fetal status.

However, there are limited reports on the management of pitNETs during pregnancy. In this study, we reviewed 10 cases that were treated under multidisciplinary team (MDT) decisions. We meticulously present clinical data, maternal and fetal management strategies, insights from multidisciplinary consultations, follow-up findings, modes of fetal delivery, and other pertinent information. The primary goal of our study is to summarize the successful clinical experiences of MDT as a reference.

## 2. Materials and Methods

### 2.1. Patient Enrollment and Ethics

A retrospective analysis was conducted on ten pregnant patients with pitNETs who presented with visual disturbances during pregnancy and were admitted to the neurosurgery department of Peking Union Medical College Hospital (PUMCH) between January 1995 and July 2024. All patients underwent surgical intervention for the treatment of pitNETs during the course of their pregnancy. The data set included general information, clinical symptoms, imaging features, perioperative treatment, pathological classification, and postoperative follow-up. All procedures involving human participants were conducted in accordance with the ethical standards set forth by the Institutional Ethics Committee of PUMCH at the Chinese Academy of Medical Sciences and PUMCH and in accordance with the Declaration of Helsinki. Furthermore, informed consent was obtained from all participants included in the study.

### 2.2. Assessment of Visual Function

The patient's visual function was assessed, including visual acuity, visual field, and optical coherence tomography (OCT). Visual function testing included a neurophysical examination and an international standard visual acuity chart. In addition, an OCT examination was performed in consultation with an ophthalmologist. Visual acuity and visual field were assessed preoperatively and reassessed postoperatively.

### 2.3. Preoperative Considerations

**Figure 1:**
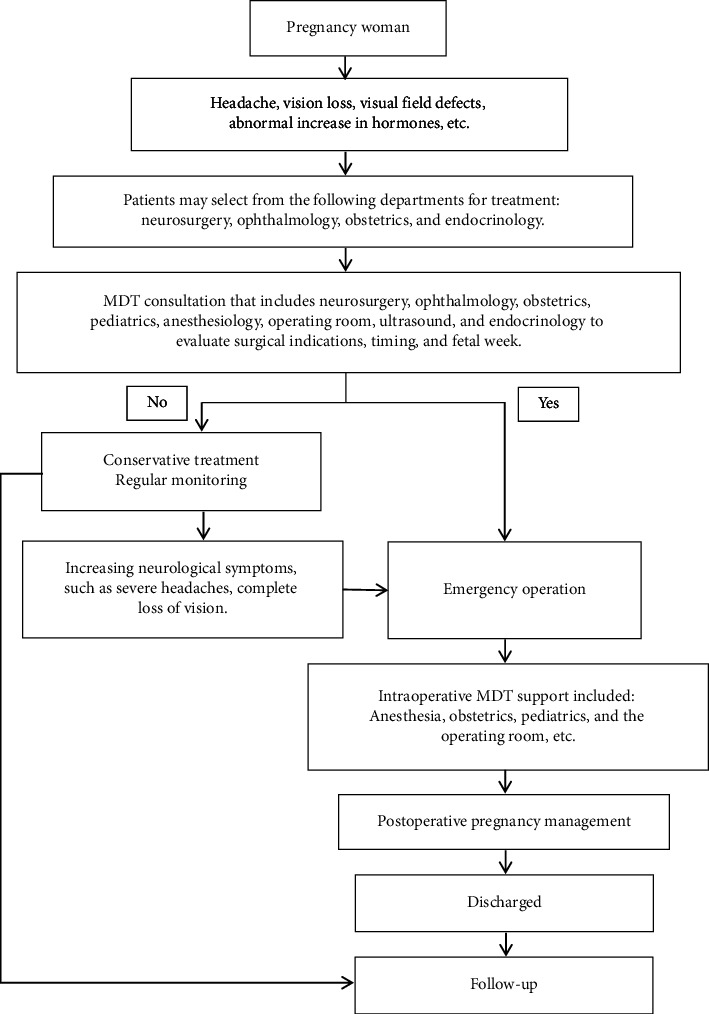
Flowchart of patient treatment options and MDT process for pitNETs during pregnancy.

For patients with pitNETs and visual impairment during pregnancy, a multidisciplinary consultation is the first step in the decision-making process for surgical intervention. The consultation involves a number of specialties, including neurosurgery, endocrinology, obstetrics, pediatrics, anesthesiology, ophthalmology, ultrasound, operating room circulating nurse, and scrub nurse. The flow diagram of the treatment option for the patient and the process of the MDT for pitNET during pregnancy are shown in Figure 1. The primary goal of is to assess the feasibility of surgery during pregnancy and to balance the potential risks and benefits to the pregnant patient.

### 2.4. Multidisciplinary Management of PitNETs During Pregnancy

Timely surgical management of pregnancy associated with pitNETs while ensuring the safety of the pregnant woman and fetus requires multidisciplinary considerations. Ophthalmology evaluates changes in vision, visual field, and OCT. Endocrinology evaluates hormone levels in the four pituitary axes. Obstetrics evaluates the surgery for contraindications, possible perioperative risk of preterm delivery, uterine contractions, vaginal bleeding, and fluid flow. The ultrasound department assists in assessing the condition of the fetus in utero. The Department of Pediatrics assesses the general condition of the pregnant woman and the risk of preterm delivery and, if preterm delivery occurs, the condition of the preterm delivery and subsequent management.

### 2.5. Statistic

Data analysis was performed using SPSS version 26.0 (IBM Corp., Armonk, NY, USA). Categorical variables were expressed as frequencies and percentages, and the chi-square test (χ^2^ test) was used to compare the groups. Continuous data were expressed as the mean ± standard deviation or median ± interquartile range for normally and non-normally distributed data, respectively. The normality of the data was analyzed by the one-sample Kolmogorov–Smirnov test, while variances were tested for homogeneity using Levene's test for equality of variances. We tested differences between groups with the *t* test when data were normally distributed and the variance was homogeneous. Paired-sample *t*-tests were used to compare changes in hormone levels preoperative and postoperative. Statistical significance was defined as a two-tailed *p* value of less than 0.05.

## 3. Results

### 3.1. Patient Demographics

A total of 124 patients with pitNETs during pregnancy who consulted our institute were screened. In 3 cases (late pregnancy), cesarean section was performed before transsphenoidal resection of pitNETs in the perioperative period. Apart from 111 patients who were treated conservatively, ten cases underwent transsphenoidal surgery and were included in the analysis. One patient was initially treated conservatively and transferred to transsphenoidal surgery due to worsening symptoms. The age of the ten patients ranged from 28 to 37 years (32.7 ± 2.9). Four patients presented with overt headache. One patient had monocular visual impairment, and the remaining nine patients had bilateral visual impairment. All patients presented with bilateral visual field loss. One case (10%) was diagnosed as prolactinoma, six cases (60%) were identified as nonfunctioning pituitary, two cases (20%) were classified as GH-secreting pituitary, and one case (10%) was diagnosed as TSH-secreting pituitary. One patient presented with pituitary apoplexy.

### 3.2. Patient Tumors and Pre- and Postoperative Pituitary Hormone Characteristics

**Figure 2:**
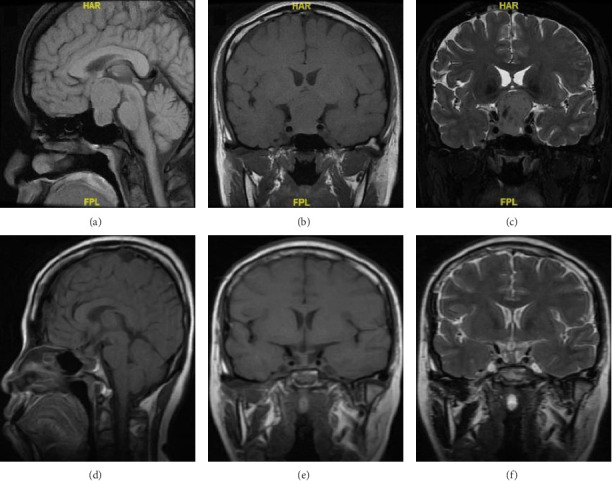
Figures (a and b) show preoperative T1 sagittal and coronal MRI images, respectively. Figures (d and e) show 16-month postoperative T1 sagittal and coronal MRI images, respectively. Figures (c and f) show preoperative and 16-month postoperative T2 coronal MRI images, respectively.

The mean value of the preoperative MRI maximum diameter of the tumor in this group was 31.2 ± 6.07 in mm. Patient 10 MRI as an example showed the lesion protruding into the sella, the pituitary stalk was not clearly visible under pressure, and the optic cross was compressed. It is closely associated with bilateral cavernous internal carotid arteries, bilateral Knosp2 grade. The short T1 signal in the posterior pituitary lobe disappeared. The pitNETs changed after surgery, the tumor was completely removed, the pituitary gland was irregularly shaped, and the pituitary stalk was thickened. Preoperative and postoperative MRI images are shown in Figure 2.

Changes in hormone levels in the 10 patients are shown in Table 1. Complete hormone monitoring was performed in all patients with prolactinoma. Prolactin levels decreased after surgery, and the difference was statistically significant (*p* < 0.05). GH levels in 5 patients with complete hormone monitoring decreased postoperatively, but without statistical significance (*p* = 0.592). IGF-1 Insulin-like growth factor-1 (IGF-1) levels decreased postoperatively in 6 patients with complete hormone monitoring, but without statistical significance (*p* = 0.598). In another 4 cases IGF-1 was not monitored. ACTH levels in the 5 patients with complete hormone monitoring decreased postoperatively, although without statistical significance (*p* = 0.247). However, in 6 patients with decreased postoperative TSH levels, the difference was not statistically significant (*p* = 0.424). All patients underwent complete tumor resection and visual acuity was restored. Patient demographics and tumor characteristics are summarized in Table 2. The results of the immunohistochemical analysis of pathological tissues are presented in Table 3.

### 3.3. Patient Obstetrical Characteristics

**Figure 3:**
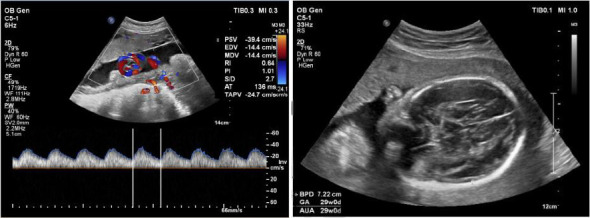
Fetal ultrasound: late intrauterine pregnancy, breech presentation (case 9).

The gestational week at the time of surgery ranged from 12 to 35 weeks with a mean of 25.5 ± 7.5 weeks. One case was identified in the first trimester (gestation < 14 weeks; 10%, 1/10), four cases in the second trimester (14 weeks ≤ gestation < 28 weeks; 40%, 4/10), and five cases in the third trimester (gestation ≥ 28 weeks; 50%, 5/10). Three patients delivered at term, two by cesarean section at 39 weeks, two at 38 weeks, one at 37 weeks, and one at 35 weeks. Nine fetuses were healthy and thriving at birth. One patient was lost to follow-up after surgery. Four patients became pregnant for the first time and two pregnant women became pregnant by in vitro fertilization (IVF). Four patients were polyparous and the other six were primiparous. Preoperative fetal ultrasound showed no significant abnormalities. All other patients showed improvement in visual acuity and perimetry as shown in Table 3. The fetal abdominal ultrasound results are shown in Figure 3.

### 3.4. Different PitNETs Treatment Options and Gestational Age

A total of 27 cases of nonfunctional pituitary adenoma were studied, 21 of which were treated conservatively and 6 of which were treated surgically. In the case of functional pituitary adenomas, a total of 94 cases were identified, with 84 cases receiving treatment with bromocriptine, 6 cases not receiving treatment, and 4 cases undergoing surgical intervention. A comparative analysis was conducted between conservative and surgical treatments for all nonfunctional and functional pitNETs, revealing a statistically significant difference between the two groups (*p* < 0.05). For all nonfunctional and one case of lactotroph, a statistical difference was observed between conservative and surgical treatments (*p* < 0.05). Similarly, for all nonfunctional and two cases of somatotroph, a statistical difference was identified between conservative and surgical treatments (*p* < 0.05). A comparative analysis was conducted between functional and nonfunctional pituitary adenomas with respect to gestational age at the time of disease onset, at the time of operation, and at the end of pregnancy. The analysis revealed no statistically significant differences (*p* > 0.05). Different pitNETs treatment options and gestational age are summarized in Table 4.

## 4. Discussion

Neurosurgeons encounter numerous challenges when treating patients with pitNETs during pregnancy. The decision between conservative or surgical treatment necessitates a comprehensive assessment. Patients with pitNETs during pregnancy require MDT collaboration to develop treatment plans, and the main departments are as follows. The first is ophthalmology, where ophthalmologists evaluate patients for visual function and optic nerve atrophy. If conservative treatment is chosen, whether the visual function can be maintained until the fetus is delivered, and the impact of the tumor on the optic nerve, whether the damaged optic nerve is reversible. The second is the endocrinology department, which evaluates hormone levels in pregnant women and whether abnormal hormone levels can be treated with medication. The third is the Department of Neurosurgery, which evaluates the benefits and risks of patients undergoing surgical treatment based on other multidisciplinary MDT evaluations, selects the surgical treatment, and performs the second round of MDT coordination and support during surgery in the departments participating in the first round of MDT consultation. Careful management during surgery to minimize complications, continued pregnancy after surgery, and follow-up care. This is the core idea of MDT in pregnancy with pitNETs. The selection of surgical intervention should also take into account the potential effects of perioperative pharmacological agents on the fetus during general anesthesia, while simultaneously prioritizing the safety and well-being of both the pregnant woman and the fetus, and optimizing the potential benefits of the surgical procedure.

PitNETs during pregnancy are rare occurrences. The patient's choice of treatment is influenced by a multitude of factors, including clinical manifestations, symptoms, the patient's personal preferences, and the level of medical expertise at the medical institution. The majority of cases are treated with medication, delayed treatment of pituitary tumors until after delivery, and, in some instances, early termination of pregnancy before transsphenoidal surgery. Transsphenoidal surgery during pregnancy is an exceedingly rare occurrence. The majority of studies and reports refer to MDT mode therapy [6–8]. The MDT collaboration was successfully employed to treat 10 patients with pitNETs during pregnancy at our center. The MDT collaboration model comprises two rounds. The initial phase is led by a neurosurgeon and includes specialists in endocrinology, anesthesiology, gynecology, obstetrics, pediatrics, ophthalmology, ultrasound, and a neurosurgery specialist nurse in the operating room. This facilitates a multidisciplinary evaluation and discussion, enabling the determination of surgical treatment following the improvement of various preoperative examinations. Subsequently, on the day of surgery, multidisciplinary collaboration during surgery is also supported by departments participating in multidisciplinary collaboration.

PitNETs during pregnancy are most often treated with drug-based conservative therapy to control the tumor hyperplasia. Prolactin microadenomas can be treated with dopamine receptor agonists, including bromocriptine and cabergoline as primary agents, and close monitoring of six sex hormones throughout pregnancy and that oral dopamine agonists are safe for infants during pregnancy [9, 10]. The study reveal that acromegaly women who became pregnant, pharmacological therapy have not been reported serious adverse effects and recommend that treatment with long-acting somatostatin receptor ligands or pegvisomant should be discontinued within 2 months before pregnancy is attempted [11, 12]. In the event of abnormalities in other hormones, it is necessary to seek the advice of a gynecological endocrinologist in order to address the issue of progesterone. Medical treatment with a dopamine agonist (most commonly bromocriptine or cabergoline) is the treatment of choice for most symptomatic prolactinomas, because these agents are highly effective at normalizing prolactin levels (∼60%–70%), restoring gonadal function (∼70%–90%) and decreasing tumor size (∼60%) [7]. And therefore, in our study, of 124 patients with pitNETs during pregnancy, 111 opted for conservative treatment and continued health monitoring, while 10 (∼8.3%) patients were accepted surgical treatment due to symptomatic considerations.

Of the 94 functional pituitary adenomas, 91 were identified as prolactin adenomas, 84 (89.4%) of which were treated with bromocriptine, while 6 (6.4%) were found to be undiagnosed after pregnancy. In one patient, the prolactin adenoma was identified in a timely manner, contraindications were excluded, and the adenoma was successfully resectable and delivered through a multidisciplinary collaborative approach. A statistically significant difference was observed between conservative and surgical treatments for all nonfunctional and functional pitNETs. Furthermore, a statistical difference was identified between conservative and surgical treatments for one case of lactotroph and two cases of somatotroph, respectively. This study found that, although there was no significant statistical difference, the gestational age at diagnosis of functional pituitary adenomas was earlier than the age of nonfunctional adenomas. Additionally, the gestational age at surgery for functional pituitary adenomas was later than the age of nonfunctional pituitary adenomas, and the gestational age at delivery for functional pituitary adenomas was earlier than the gestational age at delivery for nonfunctional pituitary adenomas. This is a matter of significant concern, given its relationship to the treatment and examination process of clinical patients during pregnancy, and the potential implications for the early detection, diagnosis, and treatment of MDT. It is therefore imperative to recognize that early detection and diagnosis, and subsequent treatment, are of paramount importance, as they serve to protect the safety of both pregnant women and fetuses. In the study population, the impact of different subtypes of pitNET on the prognosis of pregnant women and foetuses at various stages of pregnancy has not been demonstrated.

In the cases of those who have encountered severe headache, visual disturbance, pituitary apoplexy, or other significant tumor occupancy effect and related symptoms, patients should accept surgical treatment as soon as possible. Visual defects, severe headaches, abnormal hormone levels and gestational weeks are the preferred indications for surgical treatment. Multidisciplinary collaborative surgery is highly recommended [8, 11]. A total of 10 patients were accepted for surgical treatment and included in the MDTs evaluation. In their study, Hui Ping Zhong and colleagues employed two rounds of MDT collaboration. The initial round involved the administration of multiple assessments and the subsequent admission of the patient to the neurosurgical ward. The second round comprised a discussion of treatment strategies and the successful surgical treatment of four patients with sellar region lesions during pregnancy. An MDT consultation was subsequently held prior to fetal delivery [12]. Two of the 10 cases were conceived by IVF, including a patient who, at the time of the first meeting, had an elderly mother who was not easy to conceive and was very concerned about the health of the fetus and the effects of the surgery. The patient underwent transsphenoidal surgery during pregnancy and developed a deep friendship with us, which is reflected in the doctor-patient relationship, so it is recommended to include medical humanities and psychological medicine in the multidisciplinary cooperation. PitNETs during pregnancy can be effectively treated through MDT cooperation, with the procedure proceeding smoothly and postoperative recovery being favorable. For patients exhibiting inadequate response to conservative drug treatment, presenting with vision loss, visual field loss, and headache symptoms, surgical intervention following MDT evaluation is advised, and the surgical procedure is safe.

Preoperative evaluation requires an ultrasound examination of the pregnancy, which mainly evaluates the number of weeks of the fetus, the development of the fetus, the presence of intrauterine distress, and the position of the fetus in the uterus. Among 10 patients in our study, the first, second, and third trimester gestational weeks accounted for 1, 4, and 5 cases, respectively. Different weeks of gestation for transsphenoidal surgery have different effects on the fetus. Studies suggest that the operation should be performed in the second and third trimesters, and the early trimester should be delayed to the second trimester as much as possible. At the same time, the survival rate of the fetus after 27 weeks of pregnancy can reach 90% [8, 13]. In other case of brain tumors diagnosed during pregnancy Shiro et al. [14] suggest that termination of pregnancy is a good option for a brain tumor diagnosed after 34 weeks of gestation, while comprehensive treatment decisions should be made based on the severity of symptoms and the course of pregnancy in other cases. The ultrasound results also help guide the position of the pregnant woman during surgery. The operating nurses specialist MDT suggestions: First, arrange the cooperation of specialized nurses to prepare the necessary surgical materials; Second, during the operation, thermal insulation measures should be strictly taken, temperature-measuring urine tube should be placed, temperature infusion should be heated, and body surface should be heated. Third, establish effective peripheral venous access and arterial monitoring before surgery. Finally, prepare the operating room for emergency cesarean section. Combined with ultrasound examination of fetal position and pregnant woman's condition, the surgical position was rationally placed during the operation.

The MDT approach is led and initiated by the Department of Neurosurgery. Perioperative medications used refer to the Food and Drug Administration (FDA) classification index of drug pregnancy during surgery [15, 16], and fluid replenishment before surgery to fully prepare for surgery. At the same time, patients and their families should be fully informed about the risks of surgery. Anesthesiology to evaluate the teratogenic effects of anesthetics on the fetus was evaluated, and intraoperative arterial line and central venous pressure were monitored. Obstetrics assisted with fetal cardiac monitoring, prepared for preterm delivery, and prepared the pediatric unit to receive preterm infants. It is also important for the endocrinology department to evaluate perioperative hormones in pregnant women and provide treatment guidance. The MDT model involves two or more collaborative rounds and involves many specialties, including neurosurgery, endocrinology, obstetrics, pediatrics, anesthesiology, ophthalmology, ultrasound, circulating OR nurses, and scrub nurse escorts for surgical and postoperative care.

## 5. Conclusion

Management of pitNETs during pregnancy is challenging and needs comprehensive evaluation and integrated decision from the MDT. Pharmacological, nonsurgical treatment is adopted for most cases, whereas tumor resection is necessary for patients whose symptoms deteriorate. Transsphenoidal surgery under general anesthesia is safe for pregnant patients with pitNETs.

## Figures and Tables

**Table 1:** Preoperative and postoperative hormone levels.

Case ref	Preo-PRL (ng/mL) < 30.0	Preo-GH (ng/mL) < 2.0	Preo-IGF-1 (ng/mL) 43–220	Preo-ACTH (pg/mL) 7.2–63.3	Preo-Cor (ug/dL) 4.0–22.3	Preo-TSH (uIU/mL) 0.38–4.34	Posto-PRL (ng/mL) < 30.0	Posto-GH (ng/mL) < 2.0	Posto-IGF-1 (ng/mL) 43–220	Posto-ACTH (pg/mL) 7.2–63.3	Posto-Cor (ug/dL) 4.0–22.3	Posto-TSH (uIU/mL) 0.38–4.34
1	363.0^∗^	NA	NA	NA	0.25	NA	166	2	NA	NA	22.2	5.6
2	173.5	0.2	162	32	23.0	1.9	48.3	0.1	117	27.2	36.0	1.87
3	152.4	NA	NA	16	20.8	1.5	40.9	NA	NA	NA	NA	NA
4	53.0	0.9	109	20	36.1	3.0	37.8	0.1	55	8.3	11.6	0.87
5	12.6^∗^	0.17	NA	NA	23.7	1.65	7.9	0.06	NA	NA	4.1	0.46
6	113.8	0.05	311	38.7	14.3	1.2	64.1	0.1	213	10.3	11.9	0.33
7	63.8	171	951	24.8	13.8	0.86	20.9	2.9	392	5.0	22.2	0.11
8	49.6^∗^	0.5	158	10.6	33.1	1.65	NA	NA	NA	NA	NA	NA
9	22.9	0.1	188	NA	29.6	1.15	82.6	0.2	124	28.1	25.8	1.24
10	94.3^∗^	16	655	50.7	15.4	2.07	37.7	0.9	442	25.9	56.2	1.62

*Note:* Preo: Preoperation. Posto: Postoperation; Cor: Cortison; Ref: Reference.

Abbreviation: NA, Not available.

^∗^After bromocriptine treatment.

**Table 2:** General information and clinical presentation of pregnant patients who underwent transsphenoidal surgery for pitNETs.

Case	Age, ys	Sex	Headache	VL	VF	PiA	OP	DI	HT	AC	PRL	GnPn	Pregnancy	TS (mm)
1	29	F	No	Bi	BTH	No	No	No	No	No	No	G1P0	NP	32
2	36	F	Yes	Bi	BTH	No	No	No	No	No	No	G2P0	NP	20
3	34	F	No	Bi	BTH	Yes	Yes	Yes	No	No	No	G4P1	NP	30
4	32	F	No	Bi	BTH	No	No	No	Yes	No	No	G1P0	NP	29
5	37	F	Yes	Mo	BTH	No	No	No	No	No	Yes	G3P1	NP	40
6	28	F	Yes	Bi	BTH	No	No	No	No	No	No	G1P0	NP	29
7	32	F	No	Bi	BTH	Yes	No	No	No	Yes	No	G2P0	IVF	39
8	32	F	Yes	Bi	BTH	No	No	No	No	No	No	G4P1	NP	35
9	35	F	No	Bi	BTH	No	No	No	No	Yes	No	G4P1	NP	25
10	32	F	No	Bi	BTH	No	No	No	No	No	No	G1P0	IVF	33

*Note:* Bi: Binocular; BTH: Bitemporal hemianopsia; HT: Hyperthyroidism; Mo: Monocular; GnPn: Number of gravidity and parity; Acr: Acromegaly; PRL: Prolactin; PiA: Pituitary apoplexy; TS: Tumor size (maximum); F, Female.

Abbreviations: DI, Diabetes insipidus; IVF, In Vitro Fertilization; NP, natural pregnance; OP, Oculomotor paralysis; UTH, Unilateral temporal hemianopia; VF, Visual field; VL, Vision loss.

**Table 3:** Treatment and follow-up outcomes of pregnant patients who underwent surgery for pitNETs.

Case	Treatment					Pathology	Immunohistochemistry	Follow up	
	Medical therapy		Gestational weeks of disease onset	Gestational weeks of operation	Gestational weeks of pregnancy termination			Vision	Visual field
	Pre	Post							
1	Bromocriptine	No	12	12^+5d^ TSS	40 CS	NF	NA	Im	Im
2	No	No	28	32 TSS	Full term CS	NF	NA	Im	Im
3	No	No	18	22 TSS	38 CS	NF	NA	Im	Im
4	Sandostatin	No	24	26 TSS	38 CS	TSH	TSH (+), LH (+), AE1/AE3 (+), Syn (+), CgA (+), ACTH (−), GH (−), Ki-67 (index 3%), P53 (−), PRL (−);	Im	Im
5	Bromocriptine	No	26^+5d^	35 TSS	35 CS	PRL	NA	Im	Im
6	Prednisone, thyroxine	Prednisone, thyroxine	29^+2d^	30 TSS	39 CS	NF	LH (−), ACTH (+), GH (−), Ki-67 (index 5%), P53 (+), PRL (−), TSH (−), FSH (−), T-PIT (+), PIT-1 (−), CAM5.2 (+), ER (−), SSTR2 (0), SF-1 (−), MGMT (−), CgA(+);	Im	Im
7	No	Dydrogesterone, Hydrocortisone	5	15 TSS	37 CS	ACR	LH (+), ACTH (−), GH (+), Ki-67 (index 5%), P53 (−), PRL (−), TSH (−), FSH (−), T-PIT (−), PIT-1 (+), AE1/AE3 (+), CAM5.2 (+), ER (−);	Im	Im
8	Bromocriptine	Prednisone, desmopressin,	22	23 TSS	Lost to follow-up	NF	LH (+), ACTH (−), AE1/AE3 (+), GH (−), Ki-67 (index 5%), P53 (−), PRL (−), TSH (+)	Im	Im
9	No	Hydrocortisone	28^+5d^	30 TSS	39 CS	NF	LH (−), ACTH (+), GH (−), Ki-67 (index 4%), P53 (+), PRL (−), TSH (−), FSH (−), T-PIT (+), PIT-1 (+), CAM5.2 (+), ER (−), SSTR2 (PIT - I pedigree 3+, T-PIT pedigree-), SF-1 (−), MGMT (+), CgA (+)	Im	Im
10	Bromocriptine	No	30^+2d^	29 TSS	Full term CS	ACR	LH (−), ACTH (−), GH (+), Ki-67 (index 2%), P53 (−), PRL (−), TSH (−), FSH (−), T-PIT (−), PIT-1 (+), AE1/AE3 (+), CAM5.2 (+), ER(−);	Im	Im

*Note:* TSS: Transsphenoidal surgery; NF: Nonfunctional; Im: Improve.

Abbreviations: CS, Cesarean section; NA, Not available.

**Table 4:** A comparative analysis of gestational weeks and treatment options in different pitNETs.

	NF	F	*t*/*χ*^2^ value	*p* value
Treatment option			7.326	0.008^∗^
Conservation, *n* (%)	21	90		
Bromocriptine	—	84		
Untreated	—	6		
Surgery, *n* (%)	6	4		
Lactotrophs, *n* (%)	—	1	13.079	0.001^λ^
Thyrotroph, *n* (%)	—	1		
Somatotroph, *n* (%)	—	2	10.373	0.002^ϴ^
Gestational weeks				
Disease onset	161 ± 48.9	150.5 ± 79.1	0.262	0.8
Operation	174.5 ± 50.4	189.5 ± 58.5	−0.434	0.676
Pregnancy termination	274.8 ± 6.4	262.3 ± 16.0	1.622	0.149

*Note:* NF = nonfunction; F = function.

^∗^Conservative and surgical treatments were compared for all nonfunctional and functional pitNETs.

^λ^For all nonfunctional and 1 case of PRL, conservative and surgical treatments were compared.

^ϴ^For all nonfunctional and 2 case of GH, conservative and surgical treatments were compared.

## Data Availability

The data are available from the corresponding authors on reasonable request.

## Nomenclature

pitNETs: Pituitary neuroendocrine tumors
MDT: Multidisciplinary team
GH: Growth hormone
PRL: Prolactin
ACTH: Adrenocorticotrophic hormone
TSH: Thyroid-stimulating hormone
PUMCH: Peking Union Medical College Hospital
OCT: Optical coherence tomography
FDA: Food and Drug Administration
